# Self-Limiting versus Rotary Subjective Carious Tissue Removal: A Randomized Controlled Clinical Trial—2-Year Results

**DOI:** 10.3390/jcm9092738

**Published:** 2020-08-25

**Authors:** Ahmed H. Ali, Farah Ben Thani, Federico Foschi, Avijit Banerjee, Francesco Mannocci

**Affiliations:** 1 Aesthetic and Restorative Dentistry Department, College of Dentistry, University of Baghdad, Baghdad 10001, Iraq; 2 Department of Endodontics, Faculty of Dentistry, Oral & Craniofacial Sciences, King’s College London Floor 22 Tower Wing, Guy’s Dental Hospital, London SE1 9RT, UK; fbinthani@hotmail.com (F.B.T.); federico.foschi@kcl.ac.uk (F.F.); francesco.mannocci@kcl.ac.uk (F.M.); 3 Department of Therapeutic Dentistry, I. M. Sechenov First Moscow State Medical University, 119146 Moscow, Russia; 4 Conservative & MI Dentistry, Faculty of Dentistry, Oral & Craniofacial Sciences, King’s College London Floor 25 Tower Wing, Guy’s Dental Hospital, London SE1 9RT, UK; avijit.banerjee@kcl.ac.uk

**Keywords:** pulpitis, Carisolv, chemomechanical gel, rotary excavation, clinical trial, computed tomography, microscopy, caries, periapical radiograph, selective caries removal, minimally invasive dentistry

## Abstract

Background: the aim of this study was to assess the 2-year pulp survival of deep carious lesions in teeth excavated using a self-limiting protocol in a single-blind randomized controlled clinical trial. Methods: At baseline, 101 teeth with deep carious lesions in 86 patients were excavated randomly using self-limiting or control protocols. Standardized clinical examination and periapical radiographs of teeth were performed after 1- and 2-year follow-ups (REC 14/LO/0880). Results: During the 2-year period of the study, 24 teeth failed (16 and 8 at T12 and T24, respectively). Final analysis shows that 39/63 (61.9%) of teeth were deemed successful (16/33 (48.4%) and 23/30 (76.6%) in the control and experimental groups, respectively with a statistically significant difference (z score = 2.3, *p* = 0.021). Of teeth with severe and mild symptoms at T0, 42.9% and 36.7% respectively failed at T24 (*p* > 0.05). Within the self-limiting group, there was a lower success in premolars compared to molars (*p* < 0.05). Conclusion: after 2 years, there was a statistically significant higher pulp survival rate of teeth with deep carious lesions excavated using self-limiting protocols in patients with reversible pulpitis. Molars showed higher success than premolars in teeth excavated using the self-limiting protocol. There was no statistically significant association between the outcome and the severity of symptoms at T0 (ClinicalTrials.gov NCT03071588).

## 1. Introduction

Worldwide, dental caries is the most prevalent chronic condition among people [1]. Deep carious lesions are lesions approaching the pulp and radiographically penetrating three quarters of dentine thickness with a risk of pulp exposure during carious tissue removal. The pulp exposure is unavoidable when caries penetrates the entire thickness of dentine radiographically in “extremely deep carious lesions” [2]. Deep carious lesions present a challenge for dental practitioners where traditional, excessive amounts of dentine are usually removed to ensure the longevity of the subsequent restoration [3]. Management options of deep carious lesions usually vary from vital pulp therapies (VPT) such as indirect pulp capping, direct pulp capping, pulpotomies to pulpectomy followed by root canal treatment, depending on the extent of caries penetration, inflammatory condition of pulp and restorability of the tooth [2]. The absence of symptoms and maintainace of pulp sensibility and periapical health after one year are the criteria of success in VPT procedures [2]. The prognosis of treatment depends on the age of the patient, symptom severity, size of pulp exposure and type of pulp capping materials used [4]. Nowadays, the development of adhesive restorative materials in addition to a better knowledge of the caries process advocates minimally invasive cavity design and a more conservative approach, reducing the risk of pulp exposure and subsequent root canal treatment [5,6]. Minimally invasive dentistry (MID) aims at preserving and maintaining the vitality/sensibility of the pulp where possible as it has been suggested that inflamed pulps have the potential to heal [7,8]. In symptomatic or reversibly inflamed teeth with deep carious lesions, selective carious tissue removal to soft or firm dentine in one stage and stepwise excavation of the carious dentine biomass is indicated and potentially preserves pulp sensibility and function [9,10].

Chemomechanical Carisolv gel aids the retention of the affected dentine and helps eliminate the infected dentine [11,12]. In combination with an operating microscope, excavation of carious tissue using Carisolv gel has proven to maintain pulp sensibility and reduce the loss of sound tooth structure [13]. In deep carious lesions in patients presenting with symptoms of reversible pulpitis, the 1- and 2-year clinical outcomes of two alternative excavation protocols were investigated in this randomized controlled clinical trial (RCT). The self-limiting (experimental) protocol, which involved a chemomechanical selective removal of carious tissue using Carisolv gel with corresponding hand instruments combined with an operating microscope, was compared to the subjective rotary (control) protocol, which involved the use of rotary burs without magnification to remove carious tissue to the level of leathery dentine, as decided by the operator. The primary aim was to assess the pulp sensibility and periapical health using clinical and radiographic assessment after 1 and 2 years of treatment and the secondary aim was to assess if there is an association between different study variables and the outcome.

The 12-month results of this RCT showed higher success in preservation of sensibility of pulp and health of the periapical tissues in deep carious lesions excavated with the experimental protocol compared to that of the control protocol. Additionally, the odds of success for molars was four times higher than premolars. The outcome was not predicted by symptom severity. Additionally, periapical lesions detected by cone-beam computer tomography (CBCT) at the baseline visit (T0) were statistically significantly more than that detected by periapical radiographs [13].

The aims of this study were, first, to assess the 2-year clinical outcome (sensibility of pulp and health of periapical tissues) (primary outcome) of teeth with deep carious lesions excavated either by self-limiting or control protocols in patients presented with symptoms of reversible pulpitis. The secondary outcome was to assess the association between treatment outcome and age, symptom severity, cavity size, gender, arch and tooth type.

## 2. Materials and Method

### 2.1. Study Design, Sample Size and Randomization

**Figure 1:**
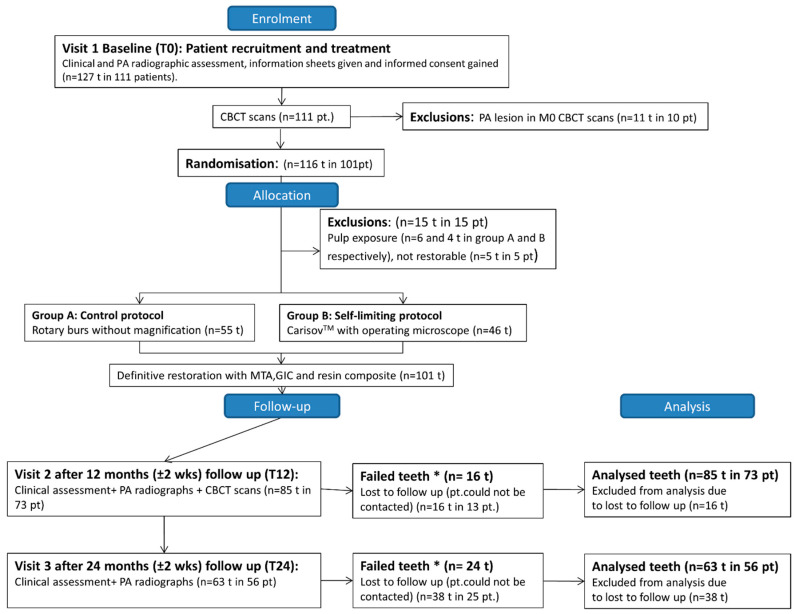
Flow diagram representing patient recruitment and follow-up. At T12 and T 24, teeth that have no response to pulp sensibility tests or have a radiolucency in the periapex or received root canal treatments categorized as failed teeth.

This single-blind 2-arm, parallel-group RCT compared the pulp survival rate of teeth with deep carious lesions excavated using a self-limiting (experimental) protocol. This involved a chemomechanical selective removal of carious tissue using Carisolv gel and corresponding hand instruments combined with an operating microscope versus the control protocol, which involved the removal of carious dentine selectively to the level of leathery dentine at the operator’s discretion using rotary burs without magnification in patients with reversible pulpitis. Ethical approval for the RCT gained from London–South East research ethics committee and registered with the National Health Service (NHS) England Research Authority (14/LO/0880, (ClinicalTrials.gov NCT03071588). A sample size of 88 restorations was calculated based on 80% power and a type I error probability with an expected difference between the 2 arms to be 20% at T12 and 10% anticipated loss of follow-ups. Location of patient recruitment (from July 2014–March 2016) and treatment was at dental clinics of King’s College London Dental Institute, London, United Kingdom. Patient information sheets were distributed, and informed written consent was obtained prior to study commencement. The tooth was the unit of block randomization performed by the Biostatistic unit Dental Institute, King’s College London. The samples were stratified according to cavity size (1, 2, or >2 walls) as a prognostic factor to be balanced during the concealed allocation of patients into each study group. Stratified random sampling was adopted for group allocation (using a random number generator). Allocation concealment was performed using a central telephone system. The patients and clinical and radiographic examiners were blinded to the protocol used. Table 1 shows the inclusion and exclusions criteria of patients. Figure 1 shows patient recruitment and follow-up flow diagram. This RCT was reported adhering to the CONSORT checklist.

### 2.2. Clinical Excavation Procedures

Operative procedures were undertaken by 30 endodontic specialist trainees, who received calibration training on carious extracted teeth using both protocols. In both excavation protocols, high-speed TA-98 air turbine handpiece (W&H Dentalwerk GmbH) with carbide and diamond burs and copious water spray was used to gain access through the enamel to the enamel–dentine junction. The soft caries-infected dentine was removed using either the control or the experimental techniques. Carbon-steel rose-head burs (Ash Instruments, Dentsply, Gloucester, UK) in a slow speed WA56A micromotor handpiece (W&H Dentalwerk Bürmoos GmbH) without using an operating microscope was used to remove caries infected dentine (CID) to the leathery tissue [14,15] in the control protocol. In the experimental group, an operating microscope (G6; Global Surgical Corporation) in combination with Carisolv gel (Rubicon Lifesciences, Gothenburg, Sweden) was used to excavate CID until no additional carious tissue was removed using the specialist hand instruments supplied.

After carious tissue excavation in both groups, a 2 mm layer of mineral trioxide aggregate (MTA) (Acteon; Pierre Rolland) was applied and condensed on the pulpal wall. After 5 min, a layer of GIC (Fuji IX; GC Corporation) was applied, and after the setting, the GIC the cavity walls were etched for 15 s using 37% phosphoric acid followed by copious water rinsing and application of a universal adhesive (Scotchbond Universal; 3M Oral Care) before placement of a resin composite overlying restoration (N’Durance; Septodont). At 12 and 24 months (±2 weeks), a standardized clinical /radiographic blinded follow-up was carried out.

### 2.3. Clinical and Radiographic Assessment

**Figure 2:**
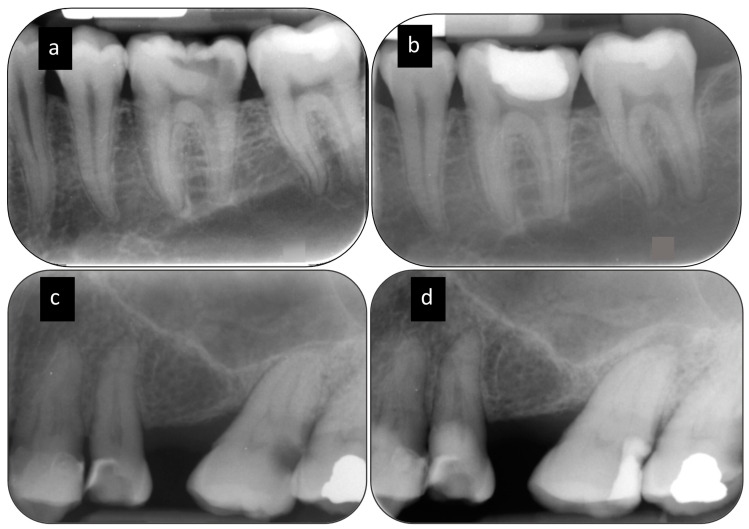
Periapical radiolucency detected by PA radiograph. (**a**) Periapical radiograph of the lower left first molar with a healthy PDL space at T0 (**b**) T24 PA radiograph shows healthy periapical tissue. (**c**) Periapical radiograph of the upper left second molar with a healthy PDL space at T0 (**d**) T24 PA radiograph shows at the periapex of the tooth a periapical radiolucency.

Clinical assessment of pulp comprised of presence/ history of pain, abscess, sinus tract and abnormal tooth mobility and performing palpation, percussion, and pulp sensibility tests. Thermal (Endo-frost; Roeko Coltène/Whaledent) and electric pulp testing (Kerr Vitality Scanner 2006; SybronEndo) performed at T0, T12 and T24. At T0 and T12, standardized periapical (PA) and cone-beam computer tomography (CBCT) radiographs were taken. At T24, only standardized PA radiographs (without CBCT radiographs) were taken. At T24, evaluation of PA radiographs performed jointly by two calibrated endodontists in a one consensus panel. The examiners were blinded to the operative protocol carried out in each tooth. After 1 month, the assessments of the PA radiographs were repeated to evaluate the intra-consensus agreement. A periapical radiolucency was considered if there was a widening of the periodontal ligament space more than two times the healthy periodontal ligament space. The consensus panel granted access to the initial PA radiographs of each case. Figure 2 shows PA radiographs detecting periapical radiolucency at T24.

### 2.4. Statistical Analysis

The primary outcome of the study was expressed as a binary variable reflecting the success or failure of the tooth in maintaining/not maintaining its sensibility at T24. In a tooth, absence of tenderness to percussion, swelling, sinus tract, spontaneous pain and periapical radiolucency and a positive response to sensibility pulp testing was regarded as a success. Descriptive statistics were used to summarize various study variables. Using IBMSPSS Statistics version 24 (IBM, Chicago, IL, USA). Kruskal–Wallis test, Chi2, and Fisher’s exact tests were used to assess the homogeneity of proportions in a categorical variable through the groups and to assess the association of different study variables (age, symptoms severity, arch, tooth type, gender and cavity size) and the outcome (secondary outcome). A two-sample two-tailed z test was used to analyze the primary outcome. The reference level of significance was set at up to 5% (α = 0.05).

## 3. Results

At T24, 47 teeth (21 and 26 in the control and self-limiting groups, respectively) were recalled for follow-up out of a total of 69 restorations at T12 (68.1%) (excluding 16 failed teeth at T12). Thirty-eight teeth in 30 patients were lost to follow-up (22 in the control versus 16 in the experimental group). Sixty-three teeth in 56 patients (including 16 failed teeth at T12) were included in the statistical analysis. Total failed teeth was 24, 16 of which failed at T12 and 8 failed at T24 follow-up. Of the failed teeth at T24 (5 and 3 in the control and experimental groups, respectively), 2/8 (25%) teeth had undergone RCT before the T24 follow-up visit, 4/8 (50%) teeth responded positively to sensibility tests and developed a periapical radiolucency in the PA radiograph. Two teeth of eight (25%) responded negatively to sensibility tests and developed a periapical radiolucency in the PA radiograph at T24. At T24 (including 16 failed teeth at T12), the pulp survival rate was 61.9% (CI 95% 0.48–0.73) (39/63 teeth) in total. The pulp survival rate of the experimental group (23/30 teeth (76.6%) (CI 95% 0.57–0.9) was statistically significantly higher than that of the control group (16/33 teeth (48.4%) (CI 95% 0.3–0.66)) (z score = 2.30, *p* = 0.021). There was a statistically significant difference showing better results in the self-limiting group. The null hypothesis was therefore rejected.

In order to evaluate the presence of an association between age and outcome, a bivariate analysis was used to categorize the age of patients into above and below 40 years old. No significant statistical association was found between cavity size, gender or age of the patients and the outcome (*p* = 0.692, *p* = 0.929 and *p* = 0.966, respectively). There was no statistically significant association between the outcome and the severity of symptoms at T0 (*p* = 0.677). Table 2 shows a summary of the association between the outcome and the clinical measures.

The association between clinical and radiographic outcome was assessed. As shown in Table 3, 73% of cases were categorised into the same outcome from both clinical and radiographic points of view. A total of 7/63 (11.1%) had clinical success but radiographic failure. A total of 10/63 (15.9%) had radiographic success but clinical failure. Kappa’s index was obtained at 0.28, which is considered as a fair concordance.

At T24, in the control group, 5/6 (83%) of premolars failed versus 6/13 (46.2%) in the experimental group with no statistically significant difference (*p* = 0.12). In the control group, 12/27 (44.4%) of molars failed versus 1/17 (5.8%) in the self-limiting group at T24, with a statistically significant difference between groups (*p*-value = 0.006). The outcome was statistically significantly associated with the tooth type within the self-limiting group (*p* = 0.009) favoring the molars over the premolars. In total, the tooth type was statistically significantly associated with the outcome (*p* = 0.03) favoring the molars over the premolars, as shown in Table 4.

## 4. Discussion

To the best of the authors’ knowledge, this is the first clinical trial reporting on the outcome of a caries excavation procedure undertaken using a minimally invasive protocol including the use of an operative microscope and Carisolv, and it is, therefore, impossible to determine if one of the two features of the minimally invasive protocol is more relevant than the other in determining the outcome of the treatment. The study design was based on best practice in caries management, i.e., the combination of a dried, well-illuminated and magnified tooth surface. The authors intended to combine the two technical aspects of an ideal protocol (enhanced magnification and chemomechanical selective caries removal) and compare this protocol with a conventional approach used commonly by dental practitioners. The self-limiting excavation protocol showed, at 2 years, a statistically significantly higher proportion of pulp survival compared to the conventional excavation group. The reduced amount of thermal and mechanical irritation the pulp is exposed to during carious tissue excavation with the experimental protocol and the self-limiting and selective behaviour of Carisolv gel [11,16,17] may have resulted in an improved response of the pulp in the experimental group. Deep dentine caries excavation using mechanical rotary burs has been correlated with the removal of tooth structure non-selectively in addition to the mechanical/thermal irritation of the dentine–pulp complex [18,19].

Symptoms of reversible pulpitis range from mild to a severe intensity which may gravitate towards irreversible pulpitis. An ambiguous borderline exists between the intensity of presenting symptoms and the condition of the pulp [20]. Some studies, however, have shown that a higher pain intensity at presentation is associated with worse histopathosis [21,22]. The results of the present study show that presenting symptom severity was not statistically associated with the outcome after two years which were in agreement with the one-year results [13]. These results, however, conflict with those reported by previous studies which found higher failure outcomes in teeth with more severely presenting symptoms [23,24]. This is likely to be associated with the exclusion of teeth presenting with pre-operative CBCT radiolucencies at baseline from this RCT. This indicates, more objectively, the intensity of pulp inflammation, as CBCT is able to overcome the limitations of PA radiographs including their inability to detect early structural changes in the periapical area due to superimposition of three-dimensional anatomical structures into a two-dimensional image, anatomical noise and geometric distortion [25].

The total success rate in this study (both in the experimental and control groups) is 61.9%, which is lower than that obtained in (Hashem et al., 2019) (72%) after two years follow up [24]. The lower total success rate in this study is because of the low success rate in the control group (48.8%) in this study, which utilized rotary instruments for carious tissue removal. However, the success rate in the experimental group (which utilized self-limiting Carisolv gel with an operating microscope) is 76.6% after 2 years, which is comparable to that obtained after 2 years in (Hashem et al., 2019) [24], in which both arms involved the use of Carisolv to remove carious tissue in teeth with reversible pulpitis and deep carious lesions. This indicates the usefulness of the self-limiting technique during carious tissue removal in maintaining pulp health in deep carious lesions.

The excavation of carious tissue with Carisolv can retain caries-affected dentine (self-limiting) compared to a subjective rotary bur excavation, which could maximize pulp protection by leaving more remaining dentine thickness over the pulp. However, this caries-affected layer needs to be remineralized, therefore the use of MTA in such cases is recommended. Carious teeth excavated with CarisolvTM gel and capped with MTA in vitro showed an increase in mineral deposition and surface hardening in the caries-affected dentine layer up to the level of sound dentine after a storage period [26]. In addition, microbiological investigations suggest that different bacteriological prints of the remaining carious dentine between the two excavation protocols may refer to the varied ecological effect of each excavation process on the microbiome of the remaining dentine after the excavation process. The mechanical and chemical differences between the two excavation methods (rotary cutting versus abrading) may mechanically force some microorganisms from the superficial to deep carious dentine layer. In addition, chemical stresses can have an impact on the bacteriological print [27]. There were previous reports suggesting a shift in the phenotypic and genotypic diversity of the remaining dentine after various restorative treatments [28,29].

The results of this study showed that 50% (4/8) of failed teeth at T24 responded positively to sensibility tests with the development of periapical radiolucency in PA radiographs. It has been shown that the release of neuropeptides from afferent fibers causes neurogenic inflammation in pulp and periapical tissue which results in the development of periapical pathology associated with a vital pulp [30]. Additionally, the coexistence of different pulpal statuses within multi-rooted teeth could result in a false-positive response to thermal and electrical sensibility tests.

Although this trial was not powered to investigate the effect of tooth type on the outcome, at two years, in total and within the self-limiting group, a statistically significant predictor of success/failure of the treatment was tooth type where, compared to premolars, a higher odds of success for molars was observed. Similar findings were observed for the 1-year results [13]. This may be attributed to the fact that molars have a larger pulp which can provide collateral vascular and neuronal innervations to the inflamed areas. This was in agreement with a study by Hørsted et al. (1985) which found that following direct pulp capping, molars had higher success compared to premolars [31]. Additionally, in the experimental group, there was a higher success in molars compared to the control group, which emphasizes the advantage of the self-limiting protocol in preserving pulpal health (Table 4).

At 2 years, the number of surfaces of preparation/restoration and arch (Maxillary vs. mandibular) did not statistically significantly affect the outcome (Table 2), similarly, in the 1-year results, cavity size (i.e., occlusal, mesio-occlusal, disto-occlusal and mesiodisto-occlusal) did not statistically significantly affect the outcome [13]. This clinical trial was not geared to investigate the effect of cavity size on the outcome, where most of the recruited cavities were within the two-surface preparation/restoration category. It was found that single-visit treatment of teeth with deep carious lesions had a better outcome when compared to two-visit treatments [32]. A dual-layer of MTA/GIC was used because the direct placement of resin composite over partially set (weak) MTA might result in a weak mechanical/chemical interaction between them in a single-visit restoration [33].

One of the limitations of this study is the lack of use of CBCT scans after two years. The use of a PA radiograph has been suggested to obscure early periapical lesions, hence failures at two–year follow-ups may have been under-reported compared to one-year follow-up. Currently, there is no recommendation to use CBCT routinely to support diagnosis and treatment in vital pulp therapies, however, when there is a presence of inconsistent signs and symptoms, CBCT usage becomes indicated in the diagnosis of periapical health/pathosis [34]. Another limitation of this study is the increased percentage of dropouts at the two-year follow-up (38 restorations from the initial 101 restoration at baseline) due to a change in contact details, refusal to attend or death.

## 5. Conclusions

After 2 years, there is a statistically significant higher pulp survival rate of teeth with deep carious lesions excavated with chemomechanical self-limiting versus rotary subjective carious tissue excavation protocols in patients with reversible pulpitis. Molars have a higher success rate than premolars in teeth excavated with the self-limiting protocol. Additionally, molars in the self-limiting group exhibited higher success than molars in the control group.

## Figures and Tables

**Table 1:** Criteria for Inclusion and Exclusion of Patients.

Inclusion Criteria	Exclusion Criteria
Patients with good general health and over the age of 18 y. In the PA radiograph, Deep carious lesion penetrating two-thirds or more into dentine thickness. Cold and electric pulp tests’ positive response. Teeth with symptoms of reversible pulpitis. The absence of PA radiolucency in preoperative CBCT scan. Posterior teeth.	Teeth with clinical symptoms of irreversible pulpitis. The presence of fistulas or swelling. Anterior teeth. External or internal root resorption. Mobile teeth or tenderness to percussion. Pregnant women.

**Table 2:** Association between clinical outcome and clinical aspects at T24.

Clinical Aspects	*p*-Value
Age	0.966
Symptom Severity	0.677
Cavity Size	0.692
Gender	0.929
Arch	0.691

**Table 3:** Relationship between clinical outcome and radiographic outcome among successful and failed teeth at T24.

Outcome	Radiographic Outcome			Total *n* (%)	Kappa *p*-Value
Success/Failure *n* (%)		Radiographic success	Radiographic failure		0.28
	Clinical Success	39 (61.9%)	7 (11.1%)	46 (73%) ^a^	
	Clinical failure	10 (15.9%)	7 (11.1%)	17 (26.9%) ^a^	
Total *n* (%)		49 (77.8%) ^b^	14 (22.2%)^b^	63 (100%)	

*n* = number of teeth, total *n* = 63, a and b are superscript small letters represent a significant difference (*p* < 0.001) between the relavent percentges.

**Table 4:** Distribution of failed teeth according to tooth type at T24.

Outcome	Control Group *n* = 33 (%) (CI 95%)		Self-Limiting Group *n* = 30 (%) (CI 95%)		Total *n* = 63 (%) (CI 95%)	
	Premolar *n* = 6	Molar *n* = 27	Premolar *n* = 13	Molar *n* = 17	Premolar *n* = 19	Molar *n* = 44
Failure *n* (%)	5 (83%) (0.41–0.98)	12 (44.4%) (0.27–0.62) ^a^	6 (46.2%) (0.23–0.7)	1 (5.8%) (0.01–0.28) ^a^	11 (57.8%) (0.36–0.76)	13 (29.5%) (0.18–0.44)
*p*-value	*p* = 0.85		*p* = 0.009 *		*p* = 0.03 *	

*n* = number of teeth, total *n* = 63, * *p* < 0.05, a is a superscript letter represent a statistically significant difference between the relevant groups. * significant difference at *p* < 0.05.
